# Foot-and-Mouth Disease Surveillance Using Pooled Milk on a Large-Scale Dairy Farm in an Endemic Setting

**DOI:** 10.3389/fvets.2020.00264

**Published:** 2020-05-27

**Authors:** Bryony Armson, Simon Gubbins, Valérie Mioulet, Ibrahim A. Qasim, Donald P. King, Nicholas A. Lyons

**Affiliations:** ^1^Vesicular Disease Reference Laboratory, The Pirbright Institute, Surrey, United Kingdom; ^2^Boyd Orr Centre for Population and Ecosystem Health, Institute of Biodiversity, Animal Health and Comparative Medicine, College of Medical, Veterinary and Life Sciences, University of Glasgow, Glasgow, United Kingdom; ^3^Directorate of Animal Resources Services, Ministry of Environment, Water and Agriculture, Riyadh, Saudi Arabia; ^4^European Commission for the Control of Foot-and-Mouth Disease (EuFMD), Animal Production and Health Division, Food and Agriculture Organization of the United Nations, Rome, Italy

**Keywords:** foot-and-mouth disease, surveillance, pooled milk, subclinical infection, vaccination, real-time RT-PCR

## Abstract

Pooled milk is used for the surveillance of several diseases of livestock. Previous studies demonstrated the detection of foot-and-mouth disease virus (FMDV) in the milk of infected animals at high dilutions, and consequently, the collection of pooled milk samples could be used to enhance FMD surveillance. This study evaluated pooled milk for FMDV surveillance on a large-scale dairy farm that experienced two FMD outbreaks caused by the A/ASIA/G-VII and O/ME-SA/Ind-2001d lineages, despite regular vaccination and strict biosecurity practices. FMDV RNA was detected in 42 (5.7%) of the 732 pooled milk samples, and typing information was concordant with diagnostic reports of clinical disease. The FMDV positive milk samples were temporally clustered around reports of new clinical cases, but with a wider distribution. For further investigation, a model was established to predict real-time RT-PCR (rRT-PCR) C_T_ values using individual cattle movement data, clinical disease records and virus excretion data from previous experimental studies. The model explained some of the instances where there were positive results by rRT-PCR, but no new clinical cases and suggested that subclinical infection occurred during the study period. Further studies are required to investigate the effect of vaccination on FMDV excretion in milk, and to evaluate more representative sampling methods. However, the results from this pilot study indicate that testing pooled milk by rRT-PCR may be valuable for FMD surveillance and has provided evidence of subclinical virus infection in vaccinated herds that could be important in the epidemiology of FMD in endemic countries where vaccination is used.

## Introduction

Milk has been exploited for the surveillance of several pathogens of livestock including bovine viral diarrhea virus (1, 2), Schmallenburg virus (3), *Coxiella burnetti* (4), bovine respiratory syncytial virus (5), and *Neospora caninum* (6). The use of pooled milk samples has also been validated as a rapid, cost-effective approach for the routine surveillance of diseases such as brucellosis (7) and mastitis caused by *Mycoplasma* spp. (8).

Previous experiments have shown that the mammary gland is an organ that is highly susceptible to foot-and-mouth disease virus (FMDV) replication, and FMDV can be detected in milk from experimentally infected animals before, during and after the appearance of clinical signs (9–13). Additionally, it has been demonstrated that FMDV can be detected and typed by real-time reverse transcription polymerase chain reaction (rRT-PCR) assays in milk from naturally infected cattle in endemic scenarios and during an outbreak in a normally FMD-free country (9, 14). Previous studies (9, 13) have suggested that it could be possible to identify one acutely-infected milking cow in a typical-sized dairy herd (100–1,000 individuals) using milk from bulk tanks or milk tankers. This theory was based on the detection of FMDV RNA in milk samples, collected from infected cattle, that had been highly diluted over 10,000-fold in negative milk. Simulation modeling using these data (13, 15, 16) support the requirement for further research to assess the use of pooled milk as a useful tool to enhance FMD surveillance.

Collection of pooled milk at the herd level could offer a representative sampling framework for FMD surveillance on large-scale dairy farms in endemic countries. Milk is routinely collected and has several advantages over vesicular material or serum by being non-invasive and potentially less susceptible to selection bias in targeted (risk-based) surveillance. For example, the use of milk does not rely on disease reporting by farmers or veterinary professionals, and sub-clinically may be confirmed using milk which would otherwise go undetected (14).

Results from the studies mentioned above have motivated further investigations using pooled milk from different production systems in endemic settings. Saudi Arabia is an FMD endemic country in which a range of production systems exist, including nomadic and small-scale herds containing small ruminants and cattle, and large-scale dairy production systems (17). Large-scale dairy farms can house in excess of 20,000 cattle, and often keep detailed records of individual cattle health, movements, milk yields and vaccination status (18–20). In recent years, Saudi Arabia has experienced outbreaks due to viral lineages that are not normally present in this region, including the A/ASIA/G-VII and O/ME-SA/Ind-2001 lineages (21, 22). These FMD outbreaks also affected large-scale dairy farms, despite regular vaccination and strict biosecurity practices, where milk was being routinely collected as part of a herd health monitoring program (18, 20).

The aim of this study was to validate the use of pooled milk for the surveillance of FMD in large-scale dairy production systems in Saudi Arabia which would also inform potential targeted/risk-based surveillance in FMD-free countries in the event of an outbreak. The specific objectives were to (i) validate the use of pooled milk collected from a large scale dairy farm in Saudi Arabia for the detection and characterization of FMDV by real-time rRT-PCR; (ii) compare the results obtained by FMDV rRT-PCR with clinical incidence; (iii) model the predicted C_T_ values of pooled milk samples based on detailed epidemiological data available from the farm; (iv) estimate the sensitivity and specificity of this surveillance approach to assess the usefulness of pooled milk as a cost-effective, non-invasive surveillance tool.

## Materials and Methods

### Study Area and Population

The study area was a large-scale dairy farm located in central Saudi Arabia. The farm housed approximately 4,000 Holstein Friesian cattle and was organized into management houses (H). Lactating groups (*n* = 17) were milked four times a day. The farm had a fenced outer perimeter and there were no other FMD susceptible livestock or wildlife present on the farm. The study population was all cattle on the farm that were in lactating groups during the study period (10/09/2015 to 25/02/2016). The farm had electronic recording systems for monitoring individual animal health and movements. Lactating cattle were regularly vaccinated every 105 days with a killed, aqueous adjuvanted (aluminum hydroxide and saponin), NSP purified FMD vaccine (containing O Manisa, O-3039, O-PanAsia2, A Iran-05, A Saudi-95, Asia-1 Shamir, and SAT-2 virus strains) (Aftovaxpur, Boehringer Ingelheim Vetmedica GmbH, Ingelheim am Rhein, Germany) (20).

In September 2015, the farm had clinical cases of FMD due to the then emerging A/ASIA/G-VII viral lineage (21), confirmed by the OIE/FAO World Reference Laboratory for foot-and-mouth disease (WRLFMD) at The Pirbright Institute, UK. In February 2016, 3 months after the last clinical case (on 12/11/2015), new clinical cases were observed and confirmed as serotype O (ME-SA/Ind-2001d lineage), with the last recorded clinical case on 07/03/2016. All recording of clinical cases was done by farm staff supervised by veterinary surgeons employed by the farms and entered into an electronic farm recording system. The FMD case definition was any individual bovine seen with increased salivation and any of the following additional clinical signs: mouth lesions, feet lesions, teat lesions, fever, reduced feed intake, and lameness. The farm policy was to isolate new cases of FMD in a dedicated isolation facility. If the isolation facility was full, or the number of observed cases in the group exceeded ~5%, cases remained within groups. Milk from clinical cases continued to be collected along with that of the other cows in the house. Animals were moved from isolation back to the main herd either after complete recovery, or when sufficiently recovered, depending on available space in the isolation facility.

### Pooled Milk Sampling

As part of routine herd health surveillance, milk samples were collected using a proportional in-line milk sampler, designed to pull a representative sample from each house, and delivered to the farm laboratory. Throughout the study period (10/09/2015 to 25/02/2016), milk samples (*n* = 732) were collected twice weekly (between 10/09/2015 and 03/12/2015), and then weekly or on an *ad-hoc* basis (between 10/12/2015 and 25/02/2016) due to the infrequency of clinical cases, until the presumed end of the outbreak. Milk samples were collected from 17 management houses that contained lactating cows and on an *ad-hoc* basis from two houses containing cows separated due to various diseases including FMD (the “sick-cow pen”). All milk samples were labeled with the date and house identification number and were stored in a freezer at −20°C until they were shipped to The Pirbright Institute (TPI, UK) for FMDV detection.

### Laboratory Testing of Pooled Milk Samples

#### Viral Isolates

FMDV cell culture isolates were obtained from archival stocks held in the WRLFMD repository. Cell culture isolate O/SAU/1/2016 was diluted in unpasteurized whole milk, and used as a positive control for the pan-serotypic rRT-PCR assay and the serotype specific O (ME-SA/Ind-2001d lineage) rRT-PCR assay. For the serotype specific A (ASIA/G-VII lineage) rRT-PCR assay, cell culture isolate A/SAU/6/2015 was diluted in unpasteurized whole milk and used as a positive control.

#### FMDV Detection Assays

RNA extraction and the pan-serotypic rRT-PCR were carried out as previously described using an optimized method (9). Briefly, RNA extractions were carried out using the MagMAX™ Pathogen RNA/DNA Kit (Applied Biosystems®) using a sample input of 200 μL on a MagMAX™ Express 96 Extraction Robot (Applied Biosystems®) according to manufacturer's instructions. VetMAX™ Xeno™ Internal Positive Control RNA (Applied Biosystems®) was added prior to extraction. Negative extraction controls consisted of unpasteurized whole milk added to lysis buffer.

The pan-serotypic rRT-PCR assay was performed using the reagents, parameters and thermal cycling conditions previously reported (23) with primers and probes described by Callahan et al. (24). One microliter per reaction of VetMAX™ Xeno™ Internal Positive Control LIZ™ Assay (Applied Biosystems®) was also included in the reaction mix. All rRT-PCR assays were performed in duplicate using an Applied Biosystems® 7500 Fast Real-time PCR System. Any milk sample with a C_T_ value of ≤ 50 was considered positive, and was also tested in duplicate on both lineage-specific rRT-PCR assays for A/ASIA/G-VII (25) and O/ME-SA/Ind-2001d (22) using the reagents, parameters and thermal cycling conditions previously reported. Additionally, samples with amplification below the 0.2 fluorescence threshold (which therefore were not considered positive) by the pan-serotypic rRT-PCR assay (termed “inconclusive” for this study), were also tested on the lineage specific rRT-PCR assays, as lower C_T_ values have previously been observed for the A/ASIA/G-VII rRT-PCR assay when compared with the pan-serotypic rRT-PCR assay (25).

### Development of a Model to Predict FMD Virus Concentrations (C_T_ Values) in Pooled Milk

To assess the limitations of the milk sampling approach, the C_T_ values of pooled milk samples were predicted using information supplied by the farm, and from the literature. These “predicted” C_T_ values were then compared with the “observed” C_T_ values obtained by the pan-serotypic rRT-PCR assays described in the previous section. The values used for each parameter are described below.

**Figure 1:**
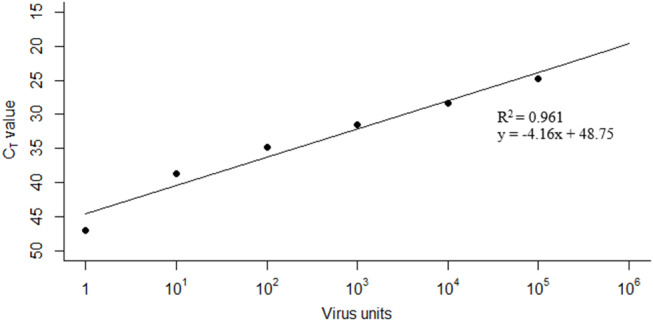
Linear regression used to predict C_T_ values from total virus unit values. Data taken from limit of detection studies performed by Armson et al. (9).

**Figure 2:**
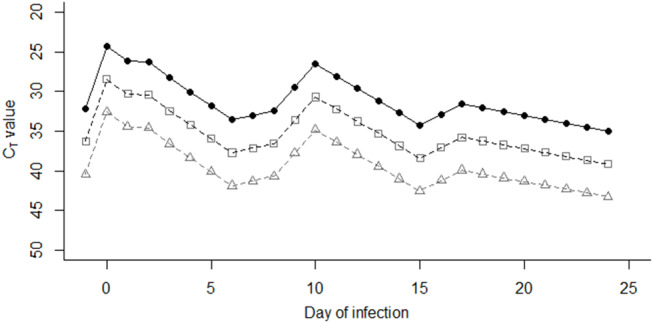
Virus unit values (U) were assigned to each stage of infection (i) between days −1 and day 24 post infection, based on mean C_T_ values of two animals in studies performed by Armson et al. (9) (closed circles). Open squares and triangles indicate the C_T_ values represented by “1/10” virus units, and “1/100” virus units, respectively.

a) **Equating C**_**T**_ **Value With the Number of Virus “Units”**
  The limit of detection of FMDV RNA in milk using the pan-serotypic rRT-PCR assay was based on a previous experimental cattle infection study (9), as this is the only study in the literature that uses the same rRT-PCR methodology. In the previous study, 10-fold serial dilutions of a whole milk sample from an infected animal gave a limit of detection of 10^−6^ (9). For this study, a viral genome unit value of 1 (subsequently referred to as a “virus unit”) was assigned to this last dilution at which FMDV RNA could be detected (i.e., 10^−6^), and subsequent virus unit values were assigned to each 10-fold dilution on a log scale (Figure 1). Linear regression was applied so that a C_T_ value could be predicted from the fit, when the total virus unit value (V) in the pooled milk was known (*R*^2^ = 0.9612, y = −4.155x + 48.75).
b) **Estimating the Number of Virus Units Excreted per Cow at Each Stage of Infection (*Ui*)**
  Using data from a previous cattle challenge study (9), FMDV RNA could be detected by the pan-serotypic rRT-PCR assay in the milk between 3 and 28 days post infection (DPI), and clinical signs were first observed at 4 DPI. As the day of infection for each cow on the large-scale farm in Saudi Arabia was unknown, the model assumed that the day clinical signs were first recorded was day [D] 0. Consequently, an excretion profile was created using the mean C_T_ values based on data collected from two in-contact animals from the challenge study (9) between D-1 to D24, subsequently referred to as the “stage of infection” (*i*) in the model (Figure 2). Missing values were interpolated, by retrieving values from the fitted line between the two nearest values. From these C_T_ values, the virus unit value (U) was predicted for each stage of infection (*i*) using the linear regression model fitted in Figure 1.
  Previous studies have described a reduced level of virus excretion in nasal fluid, saliva, and esophageal–pharyngeal fluid sample types in vaccinated vs. non-vaccinated animals (26–28). As the effect of vaccination on the duration of excretion or quantity of FMD virus in the milk is unknown, additional factors were included to account for this possibility, as milk samples in this study were collected from regularly vaccinated cattle. Data from previous studies were therefore used to inform the model (26–29), where significantly lower levels of viral excretion (by over 10^2^ copies/ml) were observed in vaccinated animals compared with unvaccinated animals. Consequently, in the model prediction for this study, three “levels” of viral excretion were adopted: “1” as described above (no vaccination), and then 10-fold reductions of “1/10” and “1/100” (Figure 2). In the model prediction, each (“1,” “1/10,” and “1/100”) virus unit value for each stage of infection (*i*) was used separately to determine the effect this change has on the resulting C_T_ value in the pooled milk sample. Additionally, the reduction was assumed to remain constant throughout the course of infection (D-1 to D24).
c) **Determining the Number of Cattle at Each Stage of Infection (*Ni*) per Sampling Date (*t*)**
  Using records of the onset of clinical signs for each cow and the movement data of individual cows between houses available from the farm, the number of cows at each stage of infection (*N*_*i*_) per sampling date (*t*) per house was calculated.
d) **Determining the Reduction in Milk Yield for Infected Cattle**
  The only milk yield data available from the farm was the average milk yield per house, per sampling date. To enable simplification of the model, it was assumed that in each house all lactating cows produced equal volumes of milk (*M*_*u*_) which was considered a reasonable assumption as cattle were placed into houses on the basis of stage of lactation and milk production.
  Due to limited studies quantifying the reduction in milk yield during FMDV infection in highly vaccinated cattle, original milk yield data from a large-scale Holstein-Friesian dairy farm in Kenya that reported a FMD outbreak in August 2012 (30, 31), were used to inform this study. For our study, the mean milk yield from 189 cattle was calculated for each 5 day period during infection (D0 to D4, D5 to D9, D10 to D14, D15 to D19, D20 to D24) as a percentage of the mean yield before infection (“normal yield”: D-10 to D-1). ANOVA and Welch two sample *T*-tests demonstrated a significant difference between D5 to D9 and normal yield (*p* = 0.001), where the value of D5 to D9 was found to be 87% of the “normal yield.” Therefore, a value of 87% of the normal yield (*M*_*i*_) was employed for each cow at stage D5–D9 of infection when determining the final number of virus units in a pooled milk sample.
e) **Determining the Final Number of Virus Units in a Pooled Milk Sample per Sampling Date [*F(t)*]**.
  Using the input parameters calculated in *a)* to *d)*, the final number of virus units in a pooled milk sample per sampling date *[F(t)]*, per house, can be calculated using the following equation:

$$\begin{array}{r} F\left(t\right)=\frac{\sum_{i=-1}^{24}M_{i}U_{i}N_{i}\left(t\right)}{\sum_{i=-1}^{24}M_{i}N_{i}\left(t\right)+M_{U}\left(H-\sum_{i=-1}^{24}N_{i}\left(t\right)\right)} \end{array}$$

Where:

- *N*_*i*_ is the number of cows at infection stage *i*
- *U*_*i*_ is the number of virus units excreted per cow at infection stage *i*
- *M*_*i*_ is the amount of milk produced by a cow in infection stage *i*
- *M*_*U*_ is the amount of milk produced by a healthy cow
- *H* is the total number of cows contributing to the milk pool

f) **Predicting C**_**T**_ **Values for Each Sampling Date (t)**
  Using the value of *F(t)* for each house the C_T_ value was predicted from the linear regression model fitted in section Equating C_T_ value with the number of virus “units.”

### Statistical Analyses

All data analyses were performed using R (version 3.5.3) (32) within the RStudio IDE (33). In order to compare the “observed” C_T_ values obtained from pooled milk samples with “predicted” C_T_ values, values were plotted for visual comparison. For each sampling date (*t*), “predicted” and “observed” C_T_ values were assigned a 0 or 1 for a negative (C_T_ of >50) or positive (C_T_ of ≤ 50) result, respectively. Additional diagnostic cut-off C_T_ values of 45 and 40 were also investigated. Contingency tables were constructed for each house, and for all houses combined using each virus unit value level (i.e., “1,” “1/10,” and “1/100”), for which sensitivity, specificity, the proportion of observed agreement (A_obs_) and the Cohen's Kappa statistic (κ) (34) were calculated. Potential clustering by management house was accounted for by using a random-effects bivariate model which was used to produce the presented sensitivity and specificity estimates (35).

## Results

### Epidemiology of the FMD Outbreaks

**Figure 3:**
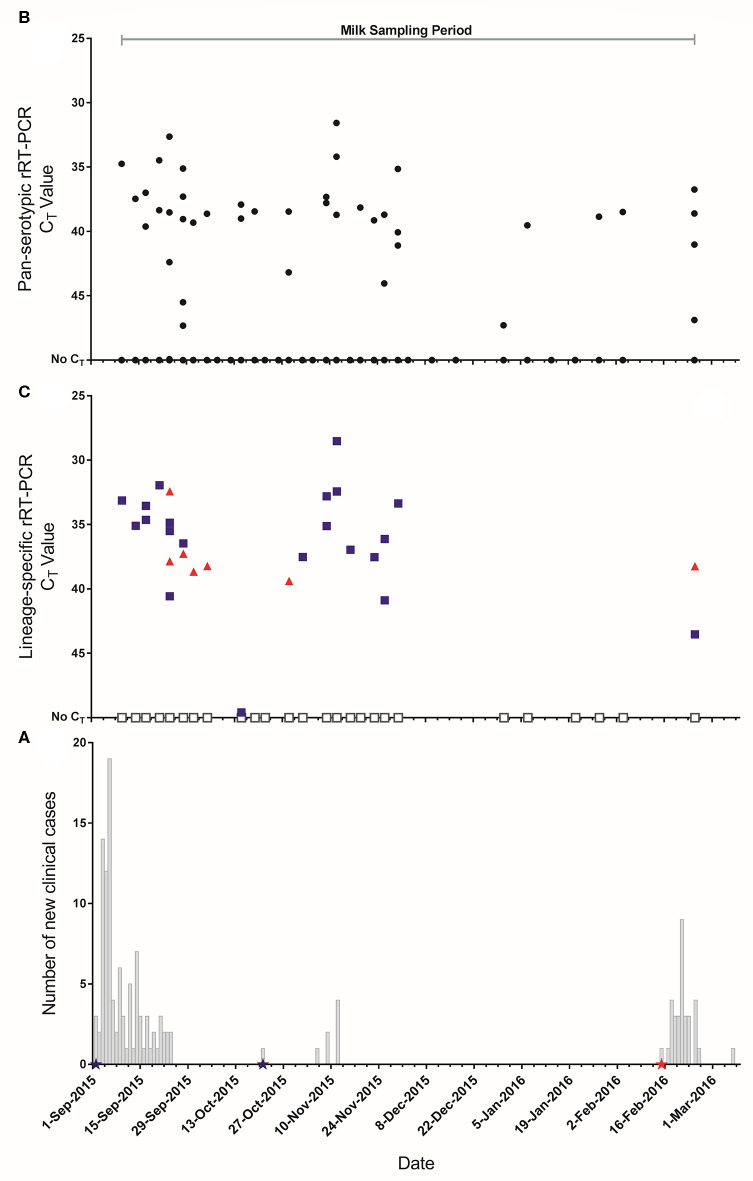
**(A)** Epidemic curves of FMD outbreaks on the farm. Stars represent dates where clinical samples (vesicular epithelium/fluid) were collected and submitted to the World Reference Laboratory for Foot-and-Mouth Disease (WRLFMD) and reported as 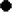: A/ASIA/G-VII, 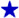: O/ME-SA/Ind-2001d. **(B)** C_T_ values from the pan-serotypic rRT-PCR assay (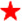) for pooled milk samples collected from 19 lactating houses in the large scale dairy farm in Saudi Arabia throughout the study period (*n* = 732). **(C)** C_T_ values for each lineage specific rRT-PCR assay for samples that tested positive (C_T_ ≤ 50), or where very low amplification was observed (below the threshold), in the pan-serotypic rRT-PCR assay. 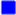: A/ASIA/G-VII. 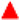: O/ME-SA/Ind-2001d. ^□^: Sample could not be typed.

Throughout the study period, the mean number of lactating cows in each house was 227 (median 237, range 44–240). Details of the farm and clinical incidence for the two FMD outbreaks are shown in Table 1. Based on the total number of cattle present on the farm, the overall incidence risk was 2.8% and 0.87% for the two separate outbreaks beginning on 02/09/2015 and 15/02/2016, respectively. The epidemic curves with corresponding sampling periods are shown in Figure 3A.

**Table 1:** Summary of outbreak data on the large-scale dairy farm in Saudi Arabia.

**Variable**		
Total number of lactating cattle during study period (approximate)	4,000	
Number of lactating houses	17	
Number of lactating animals per house^a^ (mean, median, range)	227 (237, 44–240)	
Number of lactating houses affected (%)	10 (58.8)^b^	4 (23.5)^c^
Number of clinical cases of FMD^d^	107^b^	33^c^
Overall incidence risk (number of cases/total livestock on farm) (%)	2.8^b^	0.87^c^
Date of index case	02/09/2015^b^	15/02/2016^c^

a *Calculated on milk sampling days throughout the study period*.

b *A/ASIA/GVII outbreak*.

c *O/ME-SA/Ind-2001 outbreak*.

d *Case definition used by the farm for FMD was any animal seen salivating with any of the following additional clinical signs: mouth lesions, feet lesions, teat lesions, fever, reduced feed intake, and lameness*.

### Pooled Milk

During the study period 732 milk samples were collected of which 42 (5.7%) were positive using the pan-serotypic rRT-PCR (Table 2, Figure 3B). Of these positive samples (*n* = 42), and for those not considered positive but had very low amplification below the fluorescence threshold of 0.2 (“inconclusive,” *n* = 22), 32.8% were positive by the A/ASIA/G-VII rRT-PCR assay, and 9.4% were positive by the O/ME-SA/Ind-2001d rRT-PCR assay (Figure 3C, Supplementary Data File 2). Additionally, 3.1% of the samples tested on the lineage specific assays were positive for both lineages. Of the samples that were positive on the pan-serotypic rRT-PCR assay, 19/42 (45.2%) could not be typed. Of the samples that were inconclusive on the pan-serotypic assay, 3/22 (13.6%) were positive for A/ASIA/G-VII, and 1/22 (4.5%) was positive for O/ME-SA/Ind-2001d.

**Table 2:** Summary of milk sample results for all rRT-PCR assays for the large-scale dairy farm in Saudi Arabia.

**Variable**	**Farm**
Duration of milk sampling (weeks)	25
Number of houses that milk samples were collected from	19
Number of pooled milk samples tested	732
Number positive^a^ by pan-serotypic rRT-PCR assay (%)	42 (5.7%)
Number positive^a^ by A/ASIA/G-VII rRT-PCR assay (%)	21/64^b^ (32.8%)
Number positive^a^ by O/ME-SA/Ind-2001d rRT-PCR assay (%)	6/64^b^ (9.4%)

a *Positive results are those with at least one well giving a C_T_ of ≤ 50*.

b *22 samples were considered “inconclusive” (amplification was observed below the fluorescence threshold of 0.2) and were therefore also tested by the lineage-specific rRT-PCR assays*.

### Correlation Between Epidemiological Data and FMDV RNA in Pooled Milk

Laboratory results from the pooled milk samples were directly compared against clinical data collected during the FMD outbreaks. The first period of clinical disease was seen in lactating cows between the 02/09/2015 and 24/09/2015 (*n* = 99), with two recurrences of clinical disease in a smaller number of cows in mid-October (*n* = 1) and the first half of November 2015 (*n* = 7) (Figure 3A). Clinical samples (vesicular epithelium/fluid) were collected from clinically affected animals (*n* = 3) in September and October 2015, and were characterized as belonging to the A/ASIA/G-VII lineage. Further clinical disease was recorded at the beginning of February 2016 (*n* = 33) and a clinical sample identified the strain as from the O/ME-SA/Ind-2001d lineage. Visual comparison of the epidemic curve and temporal representations of rRT-PCR results indicates some clustering of positive pooled milk samples around the occurrence of new clinical cases but with a wider distribution (Figure 3). Clustering of lineage A/ASIA/G-VII positive results can also be seen from the commencement of sampling to the end of November, concurrent with reports of this lineage from clinical samples. The clinical incidence in lactating cows over the whole study period was 3.6% (Table 1), while FMDV genome was detected in 5.7% of pooled milk samples (Table 2). A contingency table was constructed to determine the sensitivity (Se) and specificity (Sp) of the pan-serotypic rRT-PCR, using the number of new clinical cases observed on milk sample collection days for all houses sampled as the gold standard: Se = 49.3% (95% confidence interval (CI): 30.7–68.1%), Sp = 92.5% (95% CI: 90.0–94.4%) (Supplementary Data File 1).

**Figure 4:**
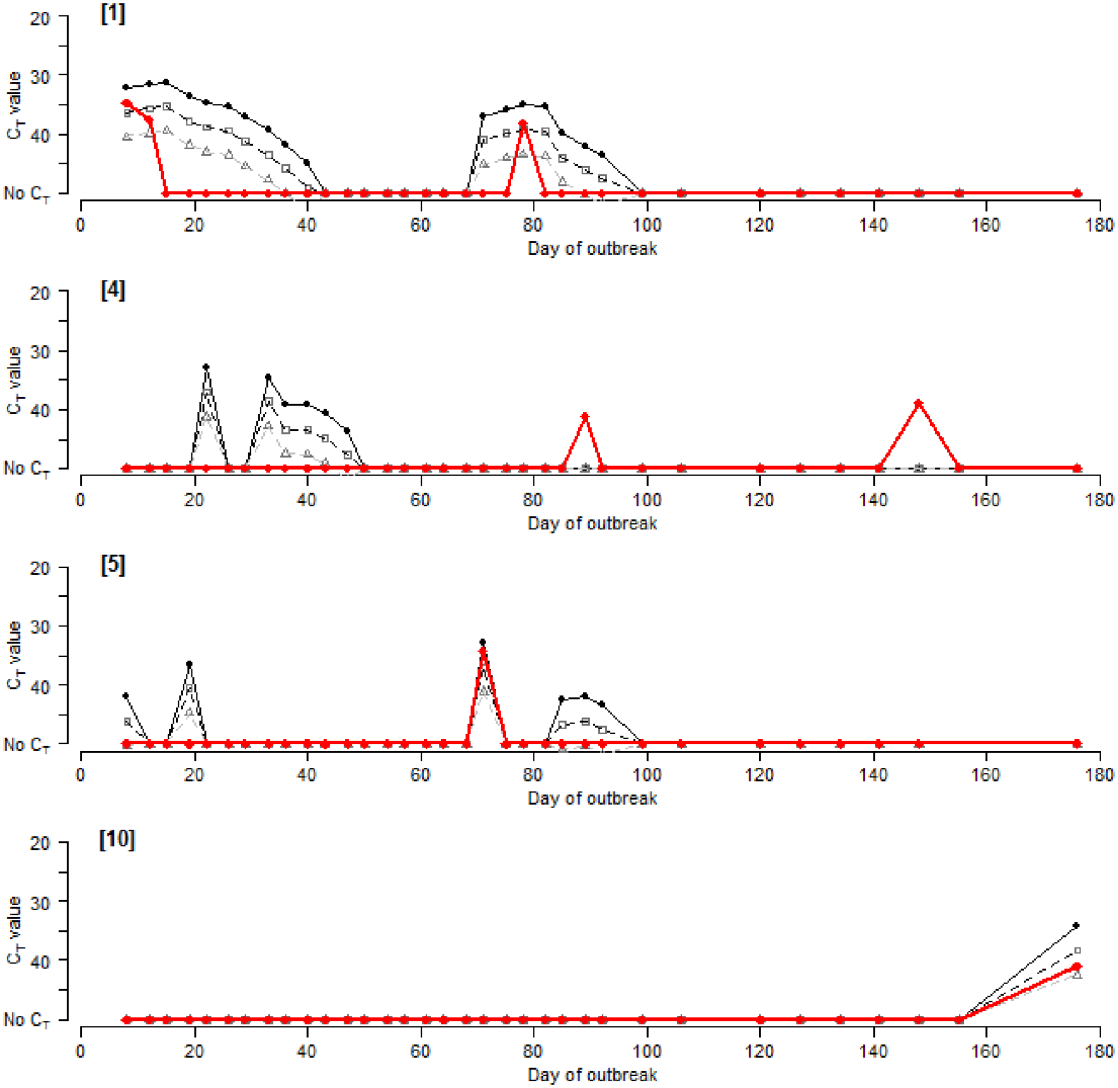
“Observed” C_T_ values for the rRT-PCR of pooled milk samples (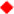) vs. “Predicted” C_T_ values at “1” viral excretion (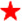), “1/10” (□) and “1/100” (△), for selected management houses 1, 4, 5, and 10. Results for the remaining houses are included in Supplementary Data Files 3, 4.

FMDV genome was detected in pooled milk in 17 out of the 19 (89.5%) sampled houses compared to 14/19 (73.7%) houses that reported clinical cases. Of the latter, 13 houses were PCR positive at some point during the outbreaks (Figure 4, Supplementary Data Files 3, 4). Furthermore, four houses were positive by rRT-PCR with no recorded clinical cases at any time during the outbreaks. There were also a total of eight samples taken where the rRT-PCR result was negative but there were new clinical cases observed on that day.

### Predicting C_T_ Values in Pooled Milk

Predicted C_T_ values were obtained for each house and compared with the observed C_T_ values from the pan-serotypic rRT-PCR (Figure 4, Supplementary Data Files 3, 4). The potential effect of reduced virus excretion that may occur due to vaccination was also investigated, where C_T_ values were predicted for the different levels of virus excretion to accommodate the possible impact of FMDV vaccination (“1,” “1/10,” and “1/100”) (Figure 4, Supplementary Data Files 3, 4). Predicted C_T_ values were not calculated for some houses due to a lack of available epidemiological data required for the analysis, or because the house was used as a quarantine pen to isolate new cases of FMD at the start of the outbreak, and therefore regular milk samples were not collected (Houses 17 and 18). Additionally, House 12 is not included in Figure 4 as both the observed and predicted results were all negative.

Visual comparison of observed vs. predicted C_T_ values revealed instances where (i) positive results were obtained for both observed and predicted, with C_T_ values that were generally comparable, (ii) positive results were obtained for the predicted values only, and (iii) positive results were obtained for the observed results only, although this was less frequent than when comparing observed C_T_ values with new clinical cases (Figure 4, Supplementary Data Files 3, 4).

The lowest predicted C_T_ values (i.e., the highest viral RNA concentration) obtained for “1,” “1/10,” and “1/100” were 30.4, 34.5, and 38.7, respectively, compared with 31.6 for the observed results. A reduction in viral excretion increased the predicted C_T_ values and, in some instances, decreased the duration for which milk samples from a house would remain positive (C_T_ ≤ 50). Additionally, applying a diagnostic cut-off value of 45 or 40 decreased the likelihood and duration of predicted positive C_T_ values. Contingency tables for all houses combined indicated that a virus excretion level of “1/10” with a diagnostic cut-off C_T_ value of 40 generated results closest to those of the observed rRT-PCR results (Se = 38.1% [95% CI: 23.2–55.6%], Sp = 95.1% [95% CI: 92.7–96.7%], A_obs_ = 0.95, *K* = 0.31) (Supplementary Data File 5). A reduction in sensitivity and increase in specificity was observed when these values were compared with estimates of sensitivity and specificity using records of new clinical cases as the “gold standard.”

## Discussion

This study aimed to expand on previous work to determine the utility of testing pooled milk by rRT-PCR as an alternative approach for FMD surveillance in vaccinated dairy herds. During the 6 month study period, 732 pooled milk samples were collected from a large-scale dairy farm housing ~4,000 cattle during an FMD outbreak.

The first objective of this study was to determine whether detection and characterization of FMDV by rRT-PCR was possible from pooled milk samples and compare these results with epidemiological data recorded during the outbreaks. This is the first study we are aware of showing that FMDV genome can be detected in milk samples from regularly vaccinated cattle using a proportional in-line milk sampler on a large-scale dairy farm. The mean C_T_ values obtained in the pan-serotypic rRT-PCR assay were high (>31), most likely due to the dilution of milk from a relatively small number of infected animals in groups of lactating cattle numbering up to 240 and collectively producing in excess of 10,000 liters per day. These results confirm the hypotheses from previous laboratory and modeling studies that suggested FMDV genome could be detected at these dilutions during outbreaks in field settings (13, 16, 36).

Lineage-specific rRT-PCR assays (22, 25) confirmed the presence of the A/ASIA/G-VII and O/ME-SA/Ind-2001d lineages in the pooled milk samples, and this was supported by reports from samples collected from clinical cases that were sent separately for laboratory testing. Reports for these samples demonstrated that the two outbreaks were caused by different FMD viral lineages, the first due to the A/ASIA/G-VII lineage, and the second the O/ME-SA/Ind-2001d lineage, both of which are thought to have emerged recently from South Asia (21, 22). The rRT-PCR results from the pooled milk samples suggest that there was a period of co-circulation or possible even co-infection with FMD viruses from these lineages. Co-infection in clinical samples from individual cattle in Saudi Arabia has been reported previously (37), though it is unknown if this occurred during the study period given that samples were taken and tested from only three clinical cases. Indeed, during this study, the collection of a variety of sample types from numerous individual animals throughout the period of infection and beyond (e.g., vesicular lesion material, blood, nasal/oral swabs and milk) may have allowed for the detection of co-infection, and may have also enabled a more thorough validation of the pooled milk surveillance approach.

Although the farm routinely vaccinated with a high potency, polyvalent FMD vaccine, it has been recently demonstrated that the serotype A components of this vaccine are not antigenically matched, and generate poor cross-protection in a potency test against A/ASIA/G-VII viruses (38). Furthermore, although individual serotype O components (such as O-3039) appear to be antigenically matched, or (O-Manisa) provide experimental protection (39) against O/ME-SA/Ind-2001d viruses, studies under field conditions (20) showed that the polyvalent vaccine used on this farm did not provide adequate heterologous cross-protection to provide full herd immunity against field viruses from the A/ASIA/G-VII and O/ME-SA/Ind-2001d lineages. This may explain why cattle still became clinically affected during the study period, albeit with a low overall incidence risk. Indeed, the A/ASIA/G-VII lineage was detected in more pooled milk samples compared to O/ME-SA/Ind-2001d during the entire study period, consistent with expected vaccine performance from respective *in vitro* vaccine-matching data and experimental studies (38, 39), A. Ludi, personal communication). The detection of a greater number of positive milk samples for the A/ASIA/G-VII lineage could also be due to the relative performance of the typing rRT-PCR assays, as in previous validation studies, lower C_T_ values for the A/ASIA/G-VII lineage typing assay have been demonstrated compared to the pan-serotypic rRT-PCR assay (indicating an increased sensitivity) (25), whilst C_T_ values for the O/ME-SA/Ind-2001d typing assay have been demonstrated to be comparable to the pan-serotypic rRT-PCR (22).

To validate the use of pooled milk for the surveillance of FMDV on this large-scale farm, pan-serotypic rRT-PCR results from the pooled milk samples were compared with the clinical incidence of FMD during the study period. At the farm level there were four temporal clusters of clinical cases with gaps of at least 15 days between these clusters. Visual appraisal of the data indicated FMDV rRT-PCR results to be generally correlated with these clusters although they showed a wider distribution around and in between the clusters of clinical cases. Comparison of the onset of individual clinical cases and the assay results on milk sampling days at the house level, revealed only 6 occasions when milk samples were positive and a new clinical case was recorded on the same day. There were also occasions when either (i) positive milk samples were obtained when there were no new clinical cases on that day, or (ii) there were new clinical cases occurring but a positive result was not observed in the milk. This resulted in a low sensitivity and moderate specificity for the pooled milk rRT-PCR assay (49.3 and 92.5%, respectively). However, this approach is limited by only comparing the assay results with the onset of new clinical cases on the sampling day which does not account for FMDV genome shedding in pre-clinical, convalescent, or subclinically infected animals.

To attempt to account for these limitations, “observed” C_T_ values obtained by the pan-serotypic rRT-PCR assays were compared with “predicted” C_T_ values for each house based on detailed epidemiological and cattle movement data from the farm, and data from recent literature. Although these results were similar, compared with the onset of clinical cases there was a reduction in sensitivity and an increase in specificity. It is likely that this may be due to the reduced number of sampling points available for the predictive analysis, due to a lack of epidemiological data available from two of the houses. It is possible that this reduced sensitivity (i.e., instances where there were positive “predicted” results but negative “observed” rRT-PCR results of the pooled milk), was due to a lower quantity and shorter duration of viral excretion in the milk of these vaccinated infected cattle, than was assumed in the model. This theory supports findings by Leeuw et al. (40) and Orsel et al. (26) who were unable to detect FMD virus in the milk of well-vaccinated cattle after challenge. However, these previous studies used a homologous or efficacious vaccine to the challenge strain and Leeuw et al. (40) only focussed on the detection of infectious live virus instead of FMDV RNA. As there are no other studies known to have considered viral excretion into the milk of vaccinated cattle, data used to inform the model was based on those studies that measured viral excretion from vaccinated and non-vaccinated animals in alternative samples such as nasal fluid, saliva, and esophageal–pharyngeal fluid (26–28). The authors acknowledge the limitation of this approach, particularly since the quantity and duration of viral excretion seemed to have a substantial impact on the likelihood of predicting a positive result in the milk. Consequently, further investigation into the effect of vaccination on viral excretion in milk is required and would enhance the predictive ability of the model.

Management practices on the farm may also have contributed to the low sensitivity of the pooled milk rRT-PCR assay. These include the inconsistent removal of clinical cases and milking practices during the study period in response to the outbreak, with the potential for increased sensitization of farmers to disease as the outbreak progressed, resulting in a decreased chance of milk from an infected cow contributing to the milk pool. Additionally, the proportional in-line sampling method may not be truly representative of all cattle in the group, as reported previously (41). Although the in-line sampler is designed to represent the whole milking, it has been demonstrated that this method may terminate sampling early (41) and milk from infected cattle may be excluded from the sample tested leading to false negative results. This may explain the low sensitivity obtained for this FMDV detection system compared with what was predicted in the model. Other methods, for example, collecting a sample from the bulk tank after thorough agitation, may be more representative (42), and could be considered for future studies.

During the study period there were also instances when there were positive rRT-PCR results in the milk samples but no new clinical cases observed, or indeed “infected” (D-1 to D24) cows present in the house that would excrete virus into the milk pool. The possibility that these “false positives” are due to laboratory contamination cannot be excluded. However, the laboratory methodology used in this study has been shown to be highly specific (data not shown), and as there were a high number of “negative” samples it is unlikely that these results are due to either laboratory contamination or non-specific amplification. Alternative explanations for this observation include spill-over of virus between houses as they were being milked (i.e., virus from an infected animal in one house may have been carried over to the milk from the subsequent house, generating false-positive results for an otherwise negative house) as there was no milk line disinfection between houses. There is also the possibility of delays in clinical case detection, sub-clinical infections or mild clinical cases that may not have been noticed by farm workers. Subclinical infections in vaccinated animals have been reported previously (43–45) and this is a possible explanation for the prolonged period between cases (up to 27 days) although it is unknown whether the outbreaks on this farm were prolonged circulation or due to new virus introductions.

This is the first study to evaluate the use of pooled milk as a surveillance sample for the detection of FMDV on large-scale dairy farms in endemic regions. This study demonstrates that rRT-PCR testing of pooled milk may be utilized for FMD surveillance and may reveal underlying sub-clinical FMD infection. More representative sampling methods should be investigated that may increase the sensitivity of this approach including investigations into the required frequency of sample collection and an exploration on how the dairy value chain may be exploited for FMD surveillance. Subsequently, this methodology could be integrated into FMD surveillance programs providing significant benefits over conventional surveillance strategies. The similarities in the farming system evaluated in this study and dairy farms in FMD-free countries highlights the potential of this surveillance approach for use in disease-free regions in the event of an incursion of FMDV, to allow rapidly identification of infected herds, tracing the source and spread of infection and to screen infected premises to ensure disease freedom.

## Data Availability Statement

All datasets analyzed for this study are included in the article/Supplementary Material.

## Ethics Statement

Ethical review and approval was not required for the animal study because samples used were collected as part of the farm's usual milking process—no change to animal's behavior/routine from normal. Written informed consent was obtained from the owners for the participation of their animals in this study.

## Author Contributions

BA carried out laboratory work, data analysis, and wrote the manuscript. NL and DK led the study design. NL assisted with data interpretation and statistical analysis and helped draft the manuscript. SG assisted with statistical analysis and formulated the model equation. IQ facilitated the collection of samples in Saudi Arabia. VM contributed reference laboratory expertise and assistance to test diagnostic samples. All authors were involved in editing and approving of the final manuscript.

## Conflict of Interest

The authors declare that the research was conducted in the absence of any commercial or financial relationships that could be construed as a potential conflict of interest.
