# Supplementary material for: Foot-and-Mouth Disease Surveillance Using Pooled Milk on a Large-Scale Dairy Farm in an Endemic Setting
Source: Front Vet Sci. 2020 May 27;7:264. doi: 10.3389/fvets.2020.00264 (PMC7267466; doi:10.3389/fvets.2020.00264)
Supplement: Supplementary file 1 [file Data_Sheet_1.PDF]

Supplementary Data File 1. Comparison of pan-serotypic rRT-PCR assay results for pooled milk with the number of new clinical cases observed on the milk sample collection day for all houses.

		New clinical cases observed		
		Positive	Negative	Total
**Pan-serotypic rRT-PCR**	Positive	6	36	42
	Negative	8	682	690
	Total	14	718	732

Se = 49.3%, Sp = 92.5%, A_{obs} = 0.94, K = 0.19
