# Supplementary material for: Foot-and-Mouth Disease Surveillance Using Pooled Milk on a Large-Scale Dairy Farm in an Endemic Setting
Source: Front Vet Sci. 2020 May 27;7:264. doi: 10.3389/fvets.2020.00264 (PMC7267466; doi:10.3389/fvets.2020.00264)
Supplement: Supplementary file 2 [file Data_Sheet_2.PDF]

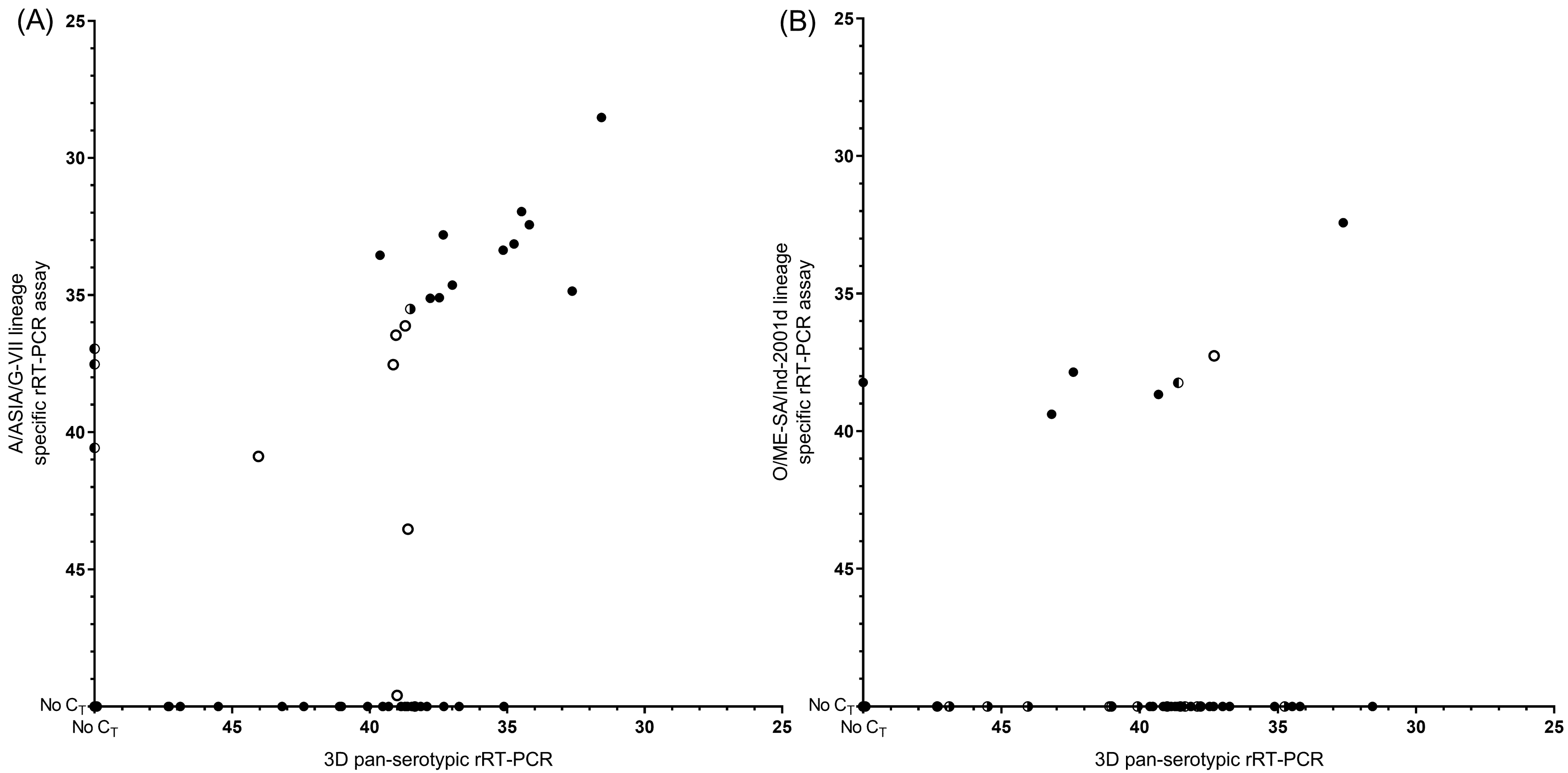

Supplementary Data File 2. Comparison of C_T values for the pan-serotypic rRT-PCR assay with the A/ASIA/G-VII lineage specific rRT-PCR assay (A) and the O/ME-SA/Ind-2001d lineage specific rRT-PCR assay (B). Average C_T values for two wells are plotted where closed points represent samples with agreement between duplicates (i.e. both negative or positive). Open points represent C_T values for individual wells where only one well was positive for each of the assays. Points with a black left half represent where only one well was positive for the lineage specific assay and both wells of the pan-serotypic assay agree (either positive or negative), while points with a black right half represent where only one well was positive for the pan-serotypic assay and both wells of the lineage specific assay agree (either positive or negative).
