# Supplementary material for: Foot-and-Mouth Disease Surveillance Using Pooled Milk on a Large-Scale Dairy Farm in an Endemic Setting
Source: Front Vet Sci. 2020 May 27;7:264. doi: 10.3389/fvets.2020.00264 (PMC7267466; doi:10.3389/fvets.2020.00264)
Supplement: Supplementary file 3 [file Data_Sheet_3.PDF]

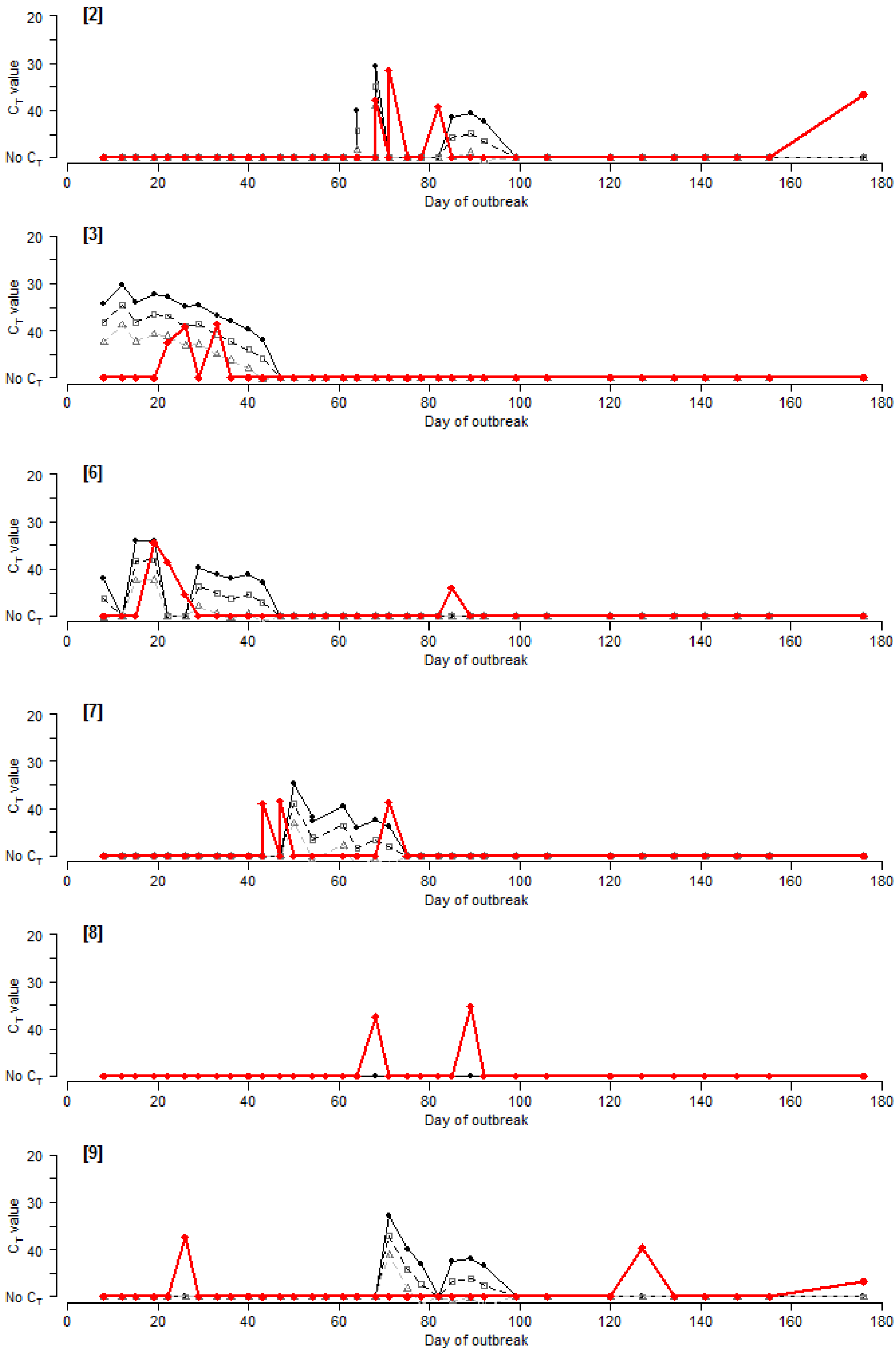

Supplementary Data File 3. 'Observed' C_T values for the rRT-PCR of pooled milk samples (♦) vs 'Predicted' C_T values at '1' viral excretion (●), '1/10' (□) and 1'100 (△), for management houses [2-3, 6-9].
