# Supplementary material for: Foot-and-Mouth Disease Surveillance Using Pooled Milk on a Large-Scale Dairy Farm in an Endemic Setting
Source: Front Vet Sci. 2020 May 27;7:264. doi: 10.3389/fvets.2020.00264 (PMC7267466; doi:10.3389/fvets.2020.00264)
Supplement: Supplementary file 4 [file Data_Sheet_4.PDF]

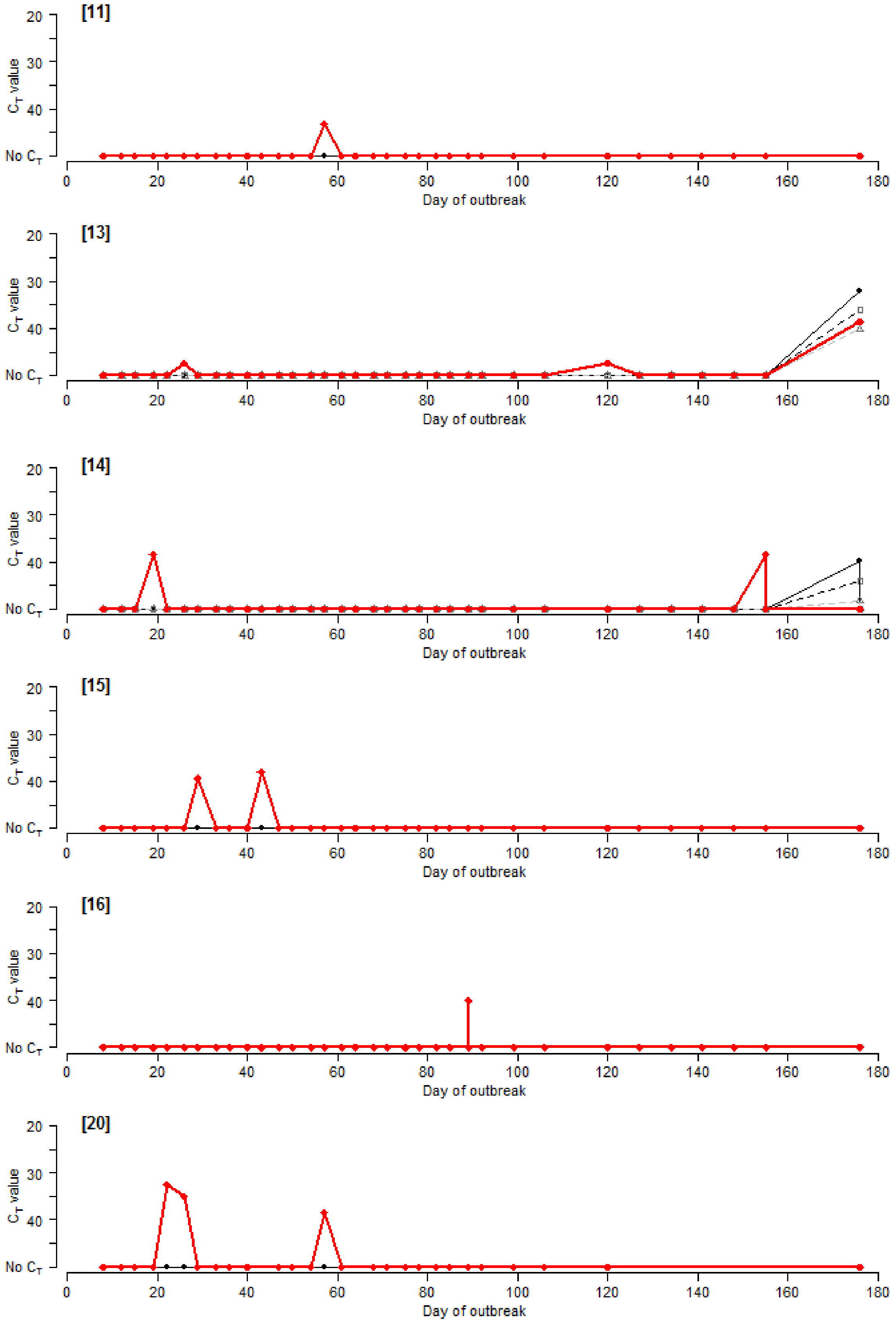

Supplementary Data File 4. 'Observed' C_T values for the rRT-PCR of pooled milk samples (♦) vs 'Predicted' C_T values at '1' viral excretion (●), '1/10' (□) and 1'100 (△), for management houses [11, 13-16, 20]. Houses 12 and 17 are not shown because they were not included in this analysis, due to the absence of clinical cases and rRT-PCR positive results in milk. House 18 was an isolation pen and not enough epidemiological data were available for analysis.
