# Supplementary material for: Foot-and-Mouth Disease Surveillance Using Pooled Milk on a Large-Scale Dairy Farm in an Endemic Setting
Source: Front Vet Sci. 2020 May 27;7:264. doi: 10.3389/fvets.2020.00264 (PMC7267466; doi:10.3389/fvets.2020.00264)
Supplement: Supplementary file 5 [file Data_Sheet_5.PDF]

Supplementary Data File 5. Comparison of observed rRT-PCR assay results for pooled milk with the predicted results number for all houses* for '1/10' virus excretion with a cut-off C_T value of 40.

		'Predicted' rRT-PCR		
		Positive	Negative	Total
**'Observed' rRT-PCR**	Positive	9	19	28
	Negative	17	657	674
	Total	26	676	702
Se = 38.1%, Sp = 95.1%, A_{obs} = 0.95, K = 0.31				

*Houses 17 and 18 were not included in the analysis due to incomplete epidemiological data.
